# Loci associated with N-glycosylation of human IgG are not associated with rheumatoid arthritis: a Mendelian randomisation study

**DOI:** 10.1136/annrheumdis-2014-207210

**Published:** 2015-09-18

**Authors:** Annie Yarwood, Sebastien Viatte, Yukinori Okada, Robert Plenge, Kazuhiko Yamamoto, BRAGGSS, RACI, Anne Barton, Deborah Symmons, Soumya Raychaudhuri, Lars Klareskog, Peter Gregersen, Jane Worthington, Steve Eyre

**Affiliations:** 1 Faculty of Medical and Human Sciences, Arthritis Research UK Centre for Genetics and Genomics, Centre for Musculoskeletal Research, Institute of Inflammation and Repair, Manchester Academic Health Science Centre, The University of Manchester, Manchester, UK; 2 Division of Rheumatology, Immunology, and Allergy, Brigham and Women's Hospital, Harvard Medical School, Boston, Massachusetts, USA; 3 Division of Genetics, Brigham and Women's Hospital, Harvard Medical School, Boston, Massachusetts, USA; 4 Program in Medical and Population Genetics, Broad Institute, Cambridge, Massachusetts, USA; 5 Department of Human Genetics and Disease Diversity, Graduate School of Medical and Dental Sciences, Tokyo Medical and Dental University, Tokyo, Japan; 6 Laboratory for Statistical Analysis, RIKEN Center for Integrative Medical Sciences, Yokohama, Japan; 7 Merck Research Laboratories, Merck & Co. Inc., Boston, Massachusetts, USA; 8 Laboratory for Autoimmune Diseases, Center for Integrative Medical Sciences, RIKEN, Yokohama, Japan; 9 Department of Allergy and Rheumatology, Graduate School of Medicine, the University of Tokyo, Tokyo, Japan; 10 NIHR Manchester Musculoskeletal Biomedical Research Unit, Central Manchester NHS Foundation Trust, Manchester Academic Health Science Centre, Manchester, UK; 11 Faculty of Medical and Human Sciences, Arthritis Research UK Centre for Epidemiology, Centre for Musculoskeletal Research, Institute of Inflammation and Repair, Manchester Academic Health Science Centre, Stopford Building, The University of Manchester, Manchester, UK; 12 Program in Medical and Population Genetics, Broad Institute of MIT and Harvard, Cambridge, Massachusetts, USA; 13 Rheumatology Unit, Department of Medicine (Solna), Karolinska Institutet, Stockholm, Sweden; 14 The Feinstein Institute for Medical Research, North Shore-Long Island Jewish Health System, Manhasset, New York, USA

**Keywords:** Autoimmunity, Gene Polymorphism, Rheumatoid Arthritis

## Abstract

**Objectives:**

A recent study identified 16 genetic variants associated with N-glycosylation of human IgG. Several of the genomic regions where these single nucleotide polymorphisms (SNPs) reside have also been associated with autoimmune disease (AID) susceptibility, suggesting there may be pleiotropy (genetic sharing) between loci controlling both N-glycosylation and AIDs. We investigated this by testing variants associated with levels of IgG N-glycosylation for association with rheumatoid arthritis (RA) susceptibility using a Mendelian randomisation study, and testing a subset of these variants in a less well-powered study of treatment response and severity.

**Methods:**

SNPs showing association with IgG N-glycosylation were analysed for association with RA susceptibility in 14 361 RA cases and 43 923 controls. Five SNPs were tested for association with response to anti-tumour necrosis factor (TNF) therapy in 1081 RA patient samples and for association with radiological disease severity in 342 patients.

**Results:**

Only one SNP (rs9296009) associated with N-glycosylation showed an association (p=6.92×10^–266^) with RA susceptibility, although this was due to linkage disequilibrium with causal human leukocyte antigen (HLA) variants. Four regions of the genome harboured SNPs associated with both traits (shared loci); although statistical analysis indicated that the associations observed for the two traits are independent. No SNPs showed association with response to anti-TNF therapy. One SNP rs12342831 was modestly associated with Larsen score (p=0.05).

**Conclusions:**

In a large, well-powered cohort of RA patients, we show SNPs driving levels of N-glycosylation have no association with RA susceptibility, indicating colocalisation of associated SNPs are not necessarily indicative of a shared genetic background or a role for glycosylation in disease susceptibility.

## Introduction

Glycosylation, a post-translational protein modification in which a carbohydrate is attached to a hydroxyl group, was first implicated in rheumatoid arthritis (RA) when serum IgG glycosylation was associated with the disease.^1^ IgG antibodies have one conserved N-glycosylation site in the Fc portion of their heavy chains, shown to influence the structure and effector functions of IgG antibodies where distinct patterns of glycosylation on anticitrullinated protein antibodies have been detected prior to the onset of RA.^2^ In addition, a lack of total IgG Fc galactosylation has been shown to correlate with RA disease activity and severity, with levels of N-terminal glycosylation being restored by treatment with anti-tumour necrosis factor (TNF) drugs.^3^ Therefore, there is accumulating evidence and interest in the hypothesis that glycosylation plays a role in susceptibility and outcome in RA.

A recent genome-wide association analysis identified 10 confirmed loci (p<2.27×10^−9^) and 7 strongly suggestive loci (p<5×10^−8^) associated with N-glycosylation of human IgG.^4^ Several of the loci identified have previously been associated with autoimmune diseases (AIDs); therefore, it has been suggested that there may be pleiotropy (shared genetic contribution) between loci controlling N-glycosylation and loci controlling AIDs.

To investigate this theory, we have performed a Mendelian randomisation study, testing variants previously associated with levels of IgG N-glycosylation for association with RA susceptibility. We also tested a subset of these single nucleotide polymorphisms (SNPs) in a smaller cohort for any indication that they may be involved in treatment response or severity in RA. Association of the same variants that control IgG N-glycosylation with susceptibility to or outcome of RA would provide an unbiased confirmation of the hypothesis that N-glycosylation is important in RA.^5^

## Methods

### Susceptibility analysis

The samples and data analysis, described previously,^6^ were from 18 studies with a total of 14 361 RA cases and 43 923 controls of European ancestry. This provided 98% power to detect an OR=1.10 at the 0.05 significance level (α), for a SNP with minor allele frequency (MAF)=20%. SNPs showing evidence of association with IgG N-glycosylation^4^ were analysed for association with RA (table 1). Of the 17 SNPs, 16 were selected for analysis, while no data were available for 1 SNP (rs1049110) in the human leukocyte antigen (HLA) region. Association analysis consisted of logistic regression correcting for up to 10 principal components. A genome-wide association study meta-analysis was then performed using an inverse variance method assuming a fixed effects model on the effect estimates (β).

**Table 1:** Meta-analysis results, testing SNPs associated with glycosylation for association with RA

Chr	SNP	SNP position	Locus	Allele 1	Allele 2	Number of studies in meta-analysis	Number of cases	Number of controls	Frequency of allele 1	Beta coefficient	SE	p Value
3	Rs11710456	186725887	ST6GAL1	A	G	11	8875	29 367	0.28	0.017	0.02	0.44
3	Rs7652995	186722944	ST6GAL1	A	G	11	8875	29 367	0.84	0.028	0.02	0.29
5	Rs17348299	55322895	IL6ST-ANKRD55	A	C	11	8875	29 367	0.16	−0.006	0.03	0.84
***7***	***Rs6421315***	***50355207***	***IKZF1***	***G***	***C***	***18***	***14 361***	***43 923***	***0***.***61***	***0***.***05***	***0***.***01***	***0***.***003***
7	Rs1122979	150915071	ABCF2-SMARCD3	A	G	11	8875	29 367	0.12	−0.04	0.0305	0.20
***9***	***Rs12342831***	***33124872***	***B4GALT1***	***T***	***C***	***17***	***13 964***	***43 524***	***0***.***74***	−***0***.***03***	***0***.***02***	***0***.***07***
11	Rs4930561	67931761	SUV420H1	A	G	11	8875	29 367	0.49	0.04	0.02	0.03
14	Rs11847263	65775695	FUT8	T	G	11	8875	29 367	0.66	−0.006	0.02	0.77
22	Rs2186369	24170996	SMARCB1-DERL3	T	G	11	8875	29 367	0.84	0.04	0.03	0.18
***22***	***Rs909674***	***39859169***	***SYNGR1-TAB1-MGAT3-CACNA1***	***A***	***C***	***18***	***14 361***	***43 923***	***0***.***73***	−***0***.***02***	***0***.***02***	***0***.***23***
***6***	***Rs9296009***	***32114515***	***PRRT1***	***A***	***T***	***18***	***14 361***	***43 923***	***0***.***79***	−***0***.***61***	***0***.***02***	***6***.***92×10^−266^***
***6***	***Rs1049110***	***32726803***	***HLA-DQA2, HLA-DQB2***	***SNP not in data***								
***6***	***Rs404256***	***90657783***	***BACH2***	***T***	***C***	***18***	***14 361***	***43 923***	***0***.***42***	***0***.***003***	***0***.***02***	***0***.***85***
7	Rs2072209	107592198	LAMB1	A	G	17	12 841	33 416	0.93	0.03	0.03	0.35
9	Rs4878639	36099399	RECK	T	C	11	8875	29 367	0.72	−0.002	0.02	0.93
12	Rs12828421	7335217	PEX5	T	C	10	7355	18 860	0.48	0.004	0.02	0.84
17	Rs7224668	79235788	SLC38A10	T	C	11	8875	29 367	0.45	−0.02	0.02	0.24

Loci shown in bold italics have previously been associated with RA (different SNP). The LD between the glycosylation SNP and RA SNP in these regions are shown in table 2. SNP p values in the individual studies included in the meta-analysis can be found in online supplementary table S1.

Chr, chromosome; LD, linkage disequilibrium; RA, rheumatoid arthritis.

### Treatment response analysis

Genotype data were available for five of the SNPs associated with glycosylation in 1039 RA patient samples from the Biologics in Rheumatoid Arthritis Genetics and Genomics Study Syndicate (BRAGGSS).^7^ Multivariate linear regression was carried out to test the association of each SNP with response to anti-TNF treatment, measured as either absolute change in DAS28 from baseline to 6 months,^8^ or according to the 6-month response criteria defined by European League Against Rheumatism (EULAR).^9^ All regression analyses were adjusted for covariates known to be predictors of response in this cohort; baseline DAS28, baseline Health Assessment Questionnaire score, concurrent disease-modifying anti-rheumatic drug therapy and gender, as described previously.^10^ As patients in the cohort received different anti-TNF drugs, a stratified analysis was also carried out.

### Outcome/severity analysis

Lack of total IgG Fc galactosylation has been shown to correlate with RA disease activity and severity. Data were available for five SNPs in patient samples from the Norfolk Arthritis Register, a primary care-based inception cohort of patients with recent onset inflammatory polyarthritis.^11^ These were tested for association with disease severity defined as radiographic damage measured by Larsen score and erosions. A generalised linear regression model was used to analyse SNP associations with erosions as a binary variable, using generalised estimating equations (STATAs xtgee). The analysis incorporated time points up to 5 years of follow-up for all patients, and was performed for disease duration <7.5 years. Generalised linear latent and mixed modelling was used to test the association of SNPs associated with glycosylation for association with Larsen score. To account for change in the tested variable (eg, Larsen score) over time, disease duration was included in all models, along with the linear quadratic terms of disease duration to account for non-linear changes over time. The same was done for age at onset as described previously.^12^

## Results

Of the 16 SNPs previously associated with glycosylation only one SNP (rs9296009) showed association with RA (p=6.92×10^−266^); however, this SNP lies within the HLA region which confers the largest genetic association with RA. The glycosylation SNP is in modest linkage disequilibrium (LD) with the most associated variants in this region (rs660895/rs6910071 (both tag HLA-DRB1*0401) r^2^=0.29, D′=0.55) and the effect observed at the glycosylation SNP is not independent of the *HLA-DRB1* susceptibility SNPs.

The HLA association with RA can be almost completely explained by five amino acid positions, three in *HLA-DRB1*, position 9 at *HLA-B* and *HLA-DPB1.*^13^ Conditioning on these five amino acids showed no evidence of a residual association in the region. Therefore, the glycosylation SNP, rs9296009, is not the lead, causal SNP in the region and there is no evidence it is independently associated with RA susceptibility.

One other SNP (rs6421315), within the *IKZF1* gene, showed modest association with RA (p=0.003). For four non-HLA loci, which contain both a SNP associated with glycosylation and a SNP associated with RA susceptibility, we examined the extent to which these associations are likely to arise from a shared genetic signal by assessing the extent of LD between the glycosylation associated SNPs and the RA associated SNPs (table 2). No evidence of significant LD was found between the SNPs in an independent dataset of 4861 European samples with genotypes available at >55 000 SNPs, suggesting that the associations observed for the two traits at these loci are independent. Further, no evidence of association was detected to the 340 SNPs from the 17 loci that showed evidence of association to a range of glycosylation traits.

**Table 2:** Linkage disequilibrium between SNPs associated with glycosylation and SNPs in the same loci previously associated with RA

Chr	SNPs associated with glycosylation (9)	Locus	SNP associated with RA (reference)	r2	D′
5	Rs17348299	IL6ST-ANKRD55	rs71624119	0.003	0.214
			rs7731626	0.002	0.149
			Rs6859219	0.001	0.167
9	Rs12342831	B4GALT1	rs11574914 (RA locus named CCL19-CCL21)	0	0.011
22	Rs909674	SYNGR1-TAB1-MGAT3-CACNA1	rs909685 (RA locus named SYNGR1)	0.108	0.848
6	Rs404256	BACH2	rs72928038	0	0.023

Linkage disequilibrium was calculated in an independent dataset of 4861 European samples where data was available for >55 000 SNPs.

Chr, chromosome; RA, rheumatoid arthritis.

We also found no evidence association with any of the five glycosylation SNPs tested with treatment response measured by change in DAS28 or EULAR response (see online supplementary tables S1 and S2).

Stratified analysis showed a modest association between rs9296009 in the *PRRT1* locus (p=0.02) and response to etanercept (n=346) measured by change in DAS28, but not when response was measured by EULAR criteria. A modest association was also seen with a SNP in the HLA-DRB1 region (rs9268839) and response to infliximab when measured by change in DAS28 (p=0.035) (n=322) and EULAR response criteria (p=0.002) (n=330) (see online supplementary tables S1 and S2).

One SNP, rs12342831, was modestly associated with severity in patients meeting American College of Rheumatology (ACR) criteria cumulatively after 5 years (n=221) (p=0.054) (see online supplementary table S3).

## Discussion

In a large, well-powered cohort of RA patients, a Mendelian randomisation approach showed no evidence to support the hypothesis that SNPs associated with N-glycosylation of IgG are associated with susceptibility to RA.

One SNP in the *IKZF1* locus showed modest association with RA in the meta-analysis (p=0.003), although it did not remain significant after correcting for multiple testing for 16 SNPs (Bonferroni corrected p value 0.05). Interestingly, *IKZF1* knockout mice were shown to have different expression of IgG N-glycans when compared with wild type.^4^ Further, different IgG N-glycan profiles exist in patients with systemic lupus erythematosus (SLE) when compared with controls, making this locus an intriguing target for further investigation. Although this locus shows no evidence for association with RA, it is associated with SLE and other AID including type 1 diabetes (T1D).^14^ ^15^ However, there is only very low LD between the glycosylation SNP (rs6421315) and the lead SLE or T1D variants respectively (rs2366293 r2=0.04 D′=0.6, rs10272724 r2=0.001, D′=0.032) suggesting possible independence.

A previous study of 127 female RA patients, followed for 6 years, showed that patients with a higher percentage of agalactosyl IgG oligosaccharides G(O) in serum had significantly more erosions and disease activity than patients with lower levels.^3^ Therefore, we tested the association of glycosylation SNPs with both response to anti-TNF therapy and disease severity in RA patients but found no evidence to support the theory that glycosylation SNPs predict outcome. Although larger than the previous study, it should be noted that the severity analysis remained underpowered, and is a major limitation of the analysis. Hence, results should be interpreted with caution and it is recommended that investigation of the effect of these glycosylation SNPs on disease outcome should be repeated in a larger cohort.

Information on glycosylation was not available in our cohort, and therefore, we could not directly test the association of variants with glycosylation. However, the use of Mendelian randomisation in the largest sample size to date has demonstrated that SNPs associated with glycosylation are not the same as those associated with RA as previously suggested, highlighting that care should be taken when inferring causality. This does not mean that glycosylation is not involved in RA; it could be a biomarker of RA as it has been shown to appear several years before disease onset.

## Supplementary Material

Web supplement

## References

[1] Parekh RB, Dwek RA, Sutton BJ Association of rheumatoid arthritis and primary osteoarthritis with changes in the glycosylation pattern of total serum IgG. Nature 1985;316:452–7. 10.1038/316452a0 3927174

[2] Rombouts Y, Ewing E, van de Stadt LA Anti-citrullinated protein antibodies acquire a pro-inflammatory Fc glycosylation phenotype prior to the onset of rheumatoid arthritis. Ann Rheum Dis 2015;74:234–41. 10.1136/annrheumdis-2013-203565 24106048

[3] Van BK, Coppieters K, Laroy W Reversible changes in serum immunoglobulin galactosylation during the immune response and treatment of inflammatory autoimmune arthritis. Ann Rheum Dis 2009;68:1360–5. 10.1136/ard.2008.089292 18772190

[4] Lauc G, Huffman JE, Pucic M Loci associated with N-glycosylation of human immunoglobulin G show pleiotropy with autoimmune diseases and haematological cancers. PLoS Genet 2013;9:e1003225 10.1371/journal.pgen.1003225 23382691 PMC3561084

[5] Hingorani A, Humphries S Nature's randomised trials. Lancet 2003;366:1906–8. 10.1016/S0140-6736(05)67767-7 16325682

[6] Okada Y, Wu D, Trynka G Genetics of rheumatoid arthritis contributes to biology and drug discovery. Nature 2014;506:376–81. 10.1038/nature12873 24390342 PMC3944098

[7] Potter C, Hyrich KL, Tracey A Association of rheumatoid factor and anti-cyclic citrullinated peptide positivity, but not carriage of shared epitope or PTPN22 susceptibility variants with tumour necrosis factor response in rheumatoid arthritis. Ann Rheum Dis 2009;68:69–74. 10.1136/ard.2007.084715 18375541 PMC2596303

[8] Prevoo ML, van ‘t Hof MA, Kuper HH Modified disease activity scores that include twenty-eight-joint counts. Development and validation in a prospective longitudinal study of patients with rheumatoid arthritis. Arthritis Rheum 1995;38:44–8. 10.1002/art.1780380107 7818570

[9] van Gestel AM, Prevoo ML, van ‘t Hof MA Development and validation of the European League Against Rheumatism response criteria for rheumatoid arthritis. Comparison with the preliminary American College of Rheumatology and the World Health Organization/International League Against Rheumatism Criteria. Arthritis Rheum 1996;39:34–40. 10.1002/art.1780390105 8546736

[10] Hyrich KL, Watson KD, Silman AJ Predictors of response to anti-TNF-alpha therapy among patients with rheumatoid arthritis: results from the British Society for Rheumatology Biologics Register. Rheumatology (Oxford) 2006;45:1558–65. 10.1093/rheumatology/kel149 16705046

[11] Symmons DP, Silman AJ The Norfolk Arthritis Register (NOAR). Clin Exp Rheumatol 2003;21 (5 Suppl 31):S94–9. 14969058

[12] Viatte S, Plant D, Lunt M Investigation of rheumatoid arthritis genetic susceptibility markers in the early rheumatoid arthritis study further replicates the TRAF1 association with radiological damage. J Rheumatol 2013;40:144–56. 10.3899/jrheum.121034 23242182

[13] Raychaudhuri S, Sandor C, Stahl EA Five amino acids in three HLA proteins explain most of the association between MHC and seropositive rheumatoid arthritis. Nat Genet 2012;44:291–6. 10.1038/ng.1076 22286218 PMC3288335

[14] Cunninghame Graham DS, Morris DL, Bhangale TR Association of NCF2, IKZF1, IRF8, IFIH1, and TYK2 with systemic lupus erythematosus. PLoS Genet 2011;7:e1002341 10.1371/journal.pgen.1002341 22046141 PMC3203198

[15] Swafford AD, Howson JM, Davison LJ An allele of IKZF1 (Ikaros) conferring susceptibility to childhood acute lymphoblastic leukemia protects against type 1 diabetes. Diabetes 2011;60:1041–4. 10.2337/db10-0446 21270240 PMC3046822

