# Supplementary material for: Loci associated with N-glycosylation of human IgG are not associated with rheumatoid arthritis: a Mendelian randomisation study
Source: Ann Rheum Dis. 2015 Sep 18;75(1):317–20. doi: 10.1136/annrheumdis-2014-207210 (PMC4717396; doi:10.1136/annrheumdis-2014-207210)
Supplement: Web supplement [file annrheumdis-2014-207210-s1.pdf]

Supplementary table 1: Glycosylation SNPs tested for association with response to anti-TNF biologic therapy using change in DAS28 after 6 months																			
change in DAS (n=1039)					Etanercept change in DAS (n=346)					Adalimumab change in DAS (n=370)					Infliximab change in DAS (n=322)				
	β coefficient	P value	95% CI			β coefficient	P value	95% CI			β coefficient	P value	95% CI			β coefficient	P value	95% CI	
rs9296009 (PRRT1)	-0.11	0.08	-0.23	0.01	rs9296009 (PRRT1)	-0.24	0.02	-0.44	-0.04	rs9296009 (PRRT1)	0.01	0.94	-0.19	0.20	rs9296009 (PRRT1)	-0.10	0.38	-0.33	0.13
rs404256 (BACH2)	0.00	0.94	-0.11	0.12	rs404256 (BACH2)	0.04	0.67	-0.15	0.24	rs404256 (BACH2)	-0.06	0.54	-0.26	0.14	rs404256 (BACH2)	0.03	0.77	-0.19	0.26
rs6421315 (IKZF1)	-0.01	0.86	-0.13	0.11	rs6421315 (IKZF1)	-0.05	0.64	-0.24	0.15	rs6421315 (IKZF1)	0.11	0.26	-0.08	0.31	rs6421315 (IKZF1)	-0.10	0.37	-0.32	0.12
rs12342831 (B4GALT1)	-0.07	0.32	-0.20	0.06	rs12342831 (B4GALNT1)	-0.03	0.77	-0.26	0.19	rs12342831 (B4GALNT1)	-0.02	0.87	-0.23	0.19	rs12342831 (B4GALNT1)	-0.13	0.30	-0.37	0.12
rs5750823 (proxy for rs909674, SYNRG1)	-0.02	0.74	-0.15	0.11	rs5750823 (proxy for rs909674, SYNRG1)	-0.10	0.35	-0.32	0.11	rs5750823 (proxy for rs909674, SYNRG1)	-0.11	0.34	-0.33	0.12	rs5750823 (proxy for rs909674, SYNRG1)	0.15	0.20	-0.08	0.39
change in DAS (n=388)					Etanercept change in DAS (n=173)					Adalimumab change in DAS (n=50)					Infliximab change in DAS (n=165)				
	β coefficient	P value	95% CI			β coefficient	P value	95% CI			β coefficient	P value	95% CI			β coefficient	P value	95% CI	
rs762995	0.17	0.10	-0.03	0.36	rs762995	0.06	0.69	-0.22	0.33	rs762995	0.51	0.06	-0.03	1.05	rs762995	0.19	0.28	-0.15	0.53

Supplementary table 2: Glycosylation SNPs tested for association with response to anti-TNF biologic therapy using EULAR response criteria after 6 months																			
EULAR response (n=1039)					Etanercept EULAR response (n=346)					Adalimumab EULAR response (n=370)					Infliximab EULAR response (n=322)				
	Odds Ratio	P value	95% CI			Odds Ratio	P value	95% CI			Odds Ratio	P value	95% CI			Odds Ratio	P value	95% CI	
rs9296009 (PRRT1)	1.18	0.18	0.93	1.49	rs9296009 (PRRT1)	1.27	0.31	0.81	1.99	rs9296009 (PRRT1)	0.89	0.54	0.60	1.31	rs9296009 (PRRT1)	1.49	0.06	0.98	2.25
rs404256 (BACH2)	1.02	0.84	0.81	1.29	rs404256 (BACH2)	0.99	0.98	0.65	1.53	rs404256 (BACH2)	1.04	0.85	0.69	1.56	rs404256 (BACH2)	1.01	0.94	0.69	1.50
rs6421315 (IKZF1)	1.07	0.58	0.85	1.34	rs6421315 (IKZF1)	0.97	0.88	0.63	1.48	rs6421315 (IKZF1)	1.04	0.86	0.70	1.54	rs6421315 (IKZF1)	1.18	0.39	0.81	1.74
rs12342831 (B4GALT1)	1.13	0.36	0.87	1.45	rs12342831 (B4GALNT1)	1.24	0.39	0.76	2.04	rs12342831 (B4GALNT1)	0.94	0.79	0.62	1.44	rs12342831 (B4GALNT1)	1.27	0.29	0.82	1.97
rs5750823 (proxy for rs909674, SYNRG1)	0.90	0.42	0.71	1.16	rs5750823 (proxy for rs909674, SYNRG1)	1.25	0.37	0.77	2.04	rs5750823 (proxy for rs909674, SYNRG1)	1.00	0.99	0.64	1.56	rs5750823 (proxy for rs909674, SYNRG1)	0.71	0.09	0.48	1.05

EULAR response (n=388)					Etanercept EULAR response (n=173)					Adalimumab EULAR response (n=41) *					Infliximab EULAR response (n=165)				
	Odds Ratio	P value	95% CI			Odds Ratio	P value	95% CI			Odds Ratio	P value	95% CI			Odds Ratio	P value	95% CI	
rs762995 (LARGE)	0.77	0.14	0.54	1.09	rs762995 (LARGE)	0.69	0.16	0.41	1.15	rs762995 (LARGE)	0.36	0.18	0.08	1.58	rs762995 (LARGE)	0.88	0.64	0.50	1.53

* note: gender = male predicts success perfectly. Gender dropped and 9 obs not used. This situation is likely due to the small number in this cell.

Supplementary table 3: Association of glycosylation SNPs with radiographic damage measured by Larsen score over 5 years (NOAR cohort).													
		Association with Larsen score, patients fulfilling ACR criteria cumulatively after 5 years<br>(total number of patients=221; total number of X-rays=342)						Association with Larsen score, all patients (RA and IP:<br>total number of patients=224; total number of X-rays=345)					
**SNP**	**locus**	**β coefficient**	**standard error**	**z**	**p value**	**95% CI**		**β coefficient**	**standard error**	**z**	**p value**	**95% CI**	
rs5750823 (proxy for rs909674)	SYNRG1	-0.52	1.17	-0.44	0.66	-2.82	1.78	-0.56	1.17	-0.48	0.63	-2.85	1.73
rs12342831	B4ALT1	-2.35	1.22	-1.92	0.05	-4.75	0.04	-2.41	1.22	-1.97	0.05	-4.80	-0.02
rs6421315	IKZF1	-1.39	1.02	-1.36	0.17	-3.40	0.62	-1.40	1.02	-1.38	0.17	-3.40	0.59
rs404256	BACH2	2.02	1.20	1.69	0.09	-0.33	4.38	0.16	1.14	0.14	0.89	-2.08	2.40
rs9296009	PRRT1	-0.63	1.27	-0.49	0.62	-3.12	1.86	-1.66	1.28	-1.29	0.20	-4.17	0.86

Supplementary table 4: Association of glycosylation SNPs with radiographic damage measured by erosions over 5 years (NOAR cohort).													
		Association with erosions, patients fulfilling ACR criteria cumulatively after 5 years<br>(total number of patients=221; total number of X-rays =342)						Association with erosions, all patients (RA and IP:<br>total number of patients=224; total number of X-rays =345)					
SNP	locus	β coefficient	standard error	z	p value	95% CI		β coefficient	standard error	z	p value	95% CI	
rs5750823 (proxy for rs909674)	SYNRG1	-0.16	0.21	-0.78	0.44	-0.56	0.24	-0.17	0.20	-0.83	0.40	-0.57	0.23
rs12342831	B4ALT1	-0.37	0.24	-1.55	0.12	-0.84	0.10	-0.38	0.24	-1.60	0.11	-0.85	0.09
rs6421315	IKZF1	-0.19	0.20	-0.95	0.34	-0.58	0.20	-0.19	0.20	-0.95	0.34	-0.58	0.20
rs404256	BACH2	-0.04	0.22	-0.19	0.85	-0.47	0.39	-0.06	0.22	-0.29	0.77	-0.49	0.36

rs9296009	PRRT1	-0.33	0.22	-1.51	0.13	-0.77	0.10	-0.36	0.22	-1.64	0.10	-0.79	0.07
